# From housewife to health worker: touching other lives and changing my own

**DOI:** 10.9745/GHSP-D-12-00038

**Published:** 2013-03-21

**Authors:** Interview conducted by Tahir Tarar, Translated by Duaa Khalid

**Affiliations:** a Suraj Private Provider Partnership, Lower Punjab, Pakistan; b Marie Stopes Society Pakistan, Karachi, Pakistan

Shaheen Hussain, the 6th of 7 children raised in an unprivileged home in a village of Punjab, Pakistan, married when she was 18 and played the traditional role of housewife and caregiver to her children, but she felt unfulfilled on a personal level. Two years ago, she became a field-based health educator with the “Suraj” (sun) social franchise of private reproductive health care providers, established by Marie Stopes Society in 3 provinces of Pakistan. Her story below, transcribed and translated from an interview, is a testimony of how this program not only touches the lives of the women who receive the reproductive health services but also of the health educators themselves.

“I have lived in Adda Chakrala, Punjab, my whole life. I went to school till class 10, but could not continue as my parents could not afford the school fee. Instead I was married when I turned 18. As expected of me, I gave birth to my first child soon after my marriage. In a span of 10 years, I had 3 children. I was fortunate that my husband understood the importance of a small family, given our limited resources. Both of us had grown up in families that had more mouths than our parents could feed, and we had vowed not to let that happen to our own children. I took care of the house and brought up my children to the best of my ability but felt unfulfilled. I knew I could and should do more.

Two years ago my husband came home from work with news of a vacancy for the position of a ‘health educator.’ He had heard of the position from the locally elected representative/counselor. It was the first time I heard of an organization called Marie Stopes Society. I learnt they required a woman who belonged to the community, was at least matriculate so that she was able to read and write Urdu, and willing to move freely within the community on a daily basis.

Having always felt like something was missing in my life and wanting to be useful to my community, I applied for the job. I can never forget that day I was told I had been selected—that day became a turning point in my life.

I was trained with a group of other women like myself from adjoining communities. After training, I embarked on visiting homes and meeting with married women of reproductive age. I made use of my training to broach the subject of pregnancies and contraceptives. At the beginning, I often had women closing their doors on me before I had finished speaking. These women felt awkward talking to me, and talking to their husbands, regarding spacing of pregnancies and family planning.

Eventually, employing the skills taught to me during my training, my perseverance, and show of empathy, women began to let me into the home to talk. Most often, mother-in-laws of young married women sat in the room. This allowed me to not only speak to the young women but also to their mothers-in-law—the decision-makers in most cases!

Today, I am invited by women to discuss personal matters and give advice. I take great satisfaction in what I do because now I can support women of my community in making informed decisions regarding spacing of their pregnancies and contraceptive choices. Those unable to pay for services have access to quality services using vouchers we offer through Suraj.

Most importantly I have managed to address the myths and misconceptions surrounding modern contraceptives. I have also been able to provide support and counseling for method continuation or method switching. Increasingly, I have sensed a difference in spousal communication through the women I interact with.

My work is my passion. It gives me inner satisfaction to be able to support women and couples in deciding on a method to help prevent unintended pregnancies. I can see the change in the lives of so many women with safe pregnancies and healthier babies, happier families.

What I did not mention so far is the monetary benefit of my work! I am proud to be contributing to my household's income and being able to give my children better opportunities.”

## Afterword

As a field-based health educator with the Suraj social franchise, Shaheen Hussain makes door-to-door visits to households in her community, raising awareness among married women of reproductive age about the benefits of healthy timing and spacing of pregnancies for the mother, child, and entire family. She also provides information on the entire range of available modern contraceptives and promotes the Suraj facilities for their quality services. Using a poverty assessment tool, Shaheen identifies women who are unable to pay for health services and provides them with vouchers that they can redeem for free quality services at the Suraj facility.

In Pakistan, women bear, on average, 4.1 children, and only 30% use contraception.^1^ However, the total demand for family planning is far higher at 55%, with 1 in every 3 pregnancies unplanned.^1^ Failure to meet the unmet need for family planning has had adverse implications on development indicators, particularly for maternal and child health. Postpartum hemorrhage, other pregnancy complications, and the consequences of unsafe abortion are major causes of maternal deaths.^2^ At 276 maternal deaths per 100,000 live births,^1^ the maternal mortality ratio needs to be reduced by three-quarters by 2015 to realize Millennium Development Goal 5 to improve maternal health. More than 80 women die each day in Pakistan, and more than 30,000 women each year, due to preventable complications during pregnancy.^1^^,^^3^

**Figure:**
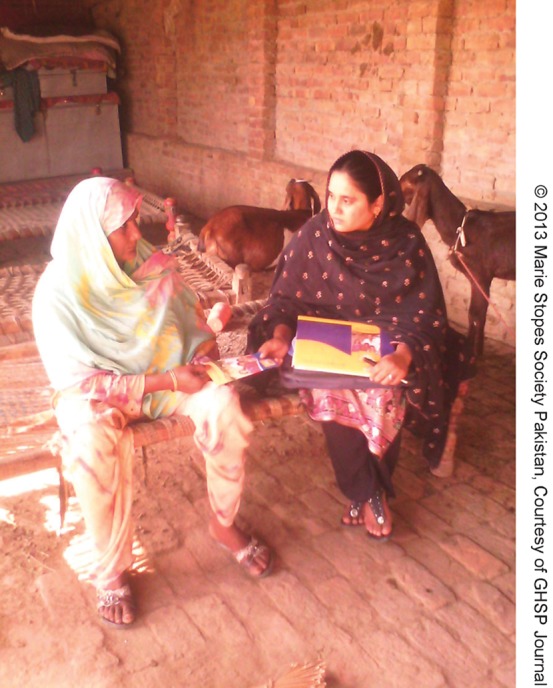
Shaheen counsels a community member about the benefits of healthy timing and spacing of pregnancies.
